# Coronary periarteritis as a rare cause of angina in a runner: hybrid PET/CT findings

**DOI:** 10.1007/s00259-026-07875-6

**Published:** 2026-04-06

**Authors:** Alexander Weissensee, Simon F. Stämpfli, Mateusz Moschkowitsch, Hannes Gruenig, Klaus Strobel, Bart W. L. De Boeck

**Affiliations:** 1 Department of Nuclear Medicine and Radiology, Luzerner Kantonsspital, University Teaching and Research Hospital, University of Lucerne, Lucerne, Switzerland; 2 Heart Center Lucerne, Luzerner Kantonsspital, University Teaching and Research Hospital, University of Lucerne, Lucerne, Switzerland; 3 Centre for Molecular Cardiology, University of Zurich, Schlieren, Switzerland

## Abstract

**Figure:**
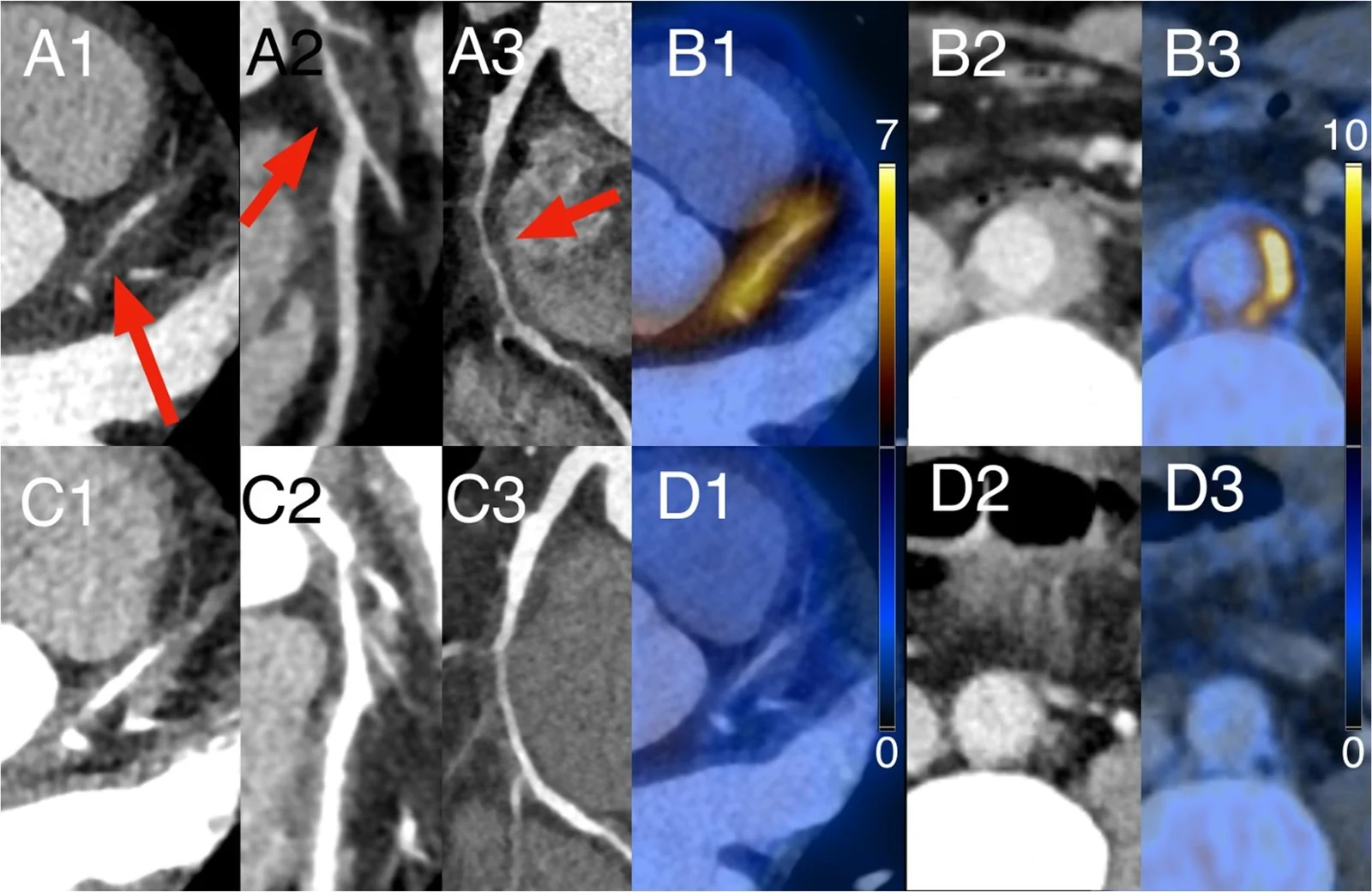

A 41-year-old former competitive athlete presented with chest pain while running. Coronary computed tomography and [^18^F]FDG PET/CT showed encasement of all major coronary vessels (A1) by intensely FDG-avid periarterial soft tissue (B1), with significant stenoses of the proximal LAD (90%, A2) and RCA (80–90%, A3). Additionally, intensely FDG-avid soft tissue partially encircled the abdominal aorta (B2–3). Baseline MRI showed 20% ischemia. Biopsy was deemed too risky. Based on the characteristic history and imaging findings, as well as the absence of atherosclerosis, the multidisciplinary team diagnosed IgG4-related disease (IgG4-RD) with involvement of the coronary arteries and abdominal aorta. Although serology was unremarkable, IgG4-RD presents with normal serum IgG4 in up to 50% of cases [1]. Therapy with glucocorticoids and rituximab was initiated [2]. Five-month follow-up [^18^F]FDG PET/CT showed complete metabolic remission (D1–3) with residual coronary stenoses (C1–3), strengthening the initial diagnosis. Follow-up MRI after 1.5 years showed 5% residual ischemia and almost complete remission of pericoronary tissue thickening. The patient regained baseline fitness within two years. Coronary arteries are involved in 1–5% of IgG4-RD cases, of which 95% show multiorgan involvement [3–5]. Imaging features are periarterial FDG-avid soft tissue thickening, with stenosis and/or aneurysms [6]. The appearance of coronary arteries wrapped by pseudotumors resembles sausages wrapped in pastry and is therefore called the “Pigs-in-a-Blanket” sign [7]. As biopsy is very risky, multimodality noninvasive imaging is pivotal in diagnosis and therapy response assessment, providing important complementary anatomic and metabolic information. While IgG4-RD with coronary involvement might be suspected on coronary CT, [^18^F]FDG PET/CT can detect other sites of involvement and assess disease activity. Correct diagnosis is crucial to prevent potentially aggravating coronary interventions and complications from untreated disease, such as myocardial infarction [8]. 

## Data Availability

Data sharing not applicable to this article as no datasets were generated or analysed during the current study.
